# Heterogeneous Nucleation of Butanol on NaCl: A Computational Study of Temperature, Humidity, Seed Charge, and Seed Size Effects

**DOI:** 10.1021/acs.jpca.0c10972

**Published:** 2021-03-31

**Authors:** Antti Toropainen, Juha Kangasluoma, Theo Kurtén, Hanna Vehkamäki, Fatemeh Keshavarz, Jakub Kubečka

**Affiliations:** † Institute for Atmospheric and Earth System Research/Physics, Faculty of Science, University of Helsinki, P.O. Box 64, Helsinki 00014, Finland; ‡ Department of Chemistry, Faculty of Science, University of Helsinki, P.O. Box 64, Helsinki 00014, Finland; § Department of Physics, School of Engineering Science, LUT University, Lappeenranta 53851, Finland

## Abstract

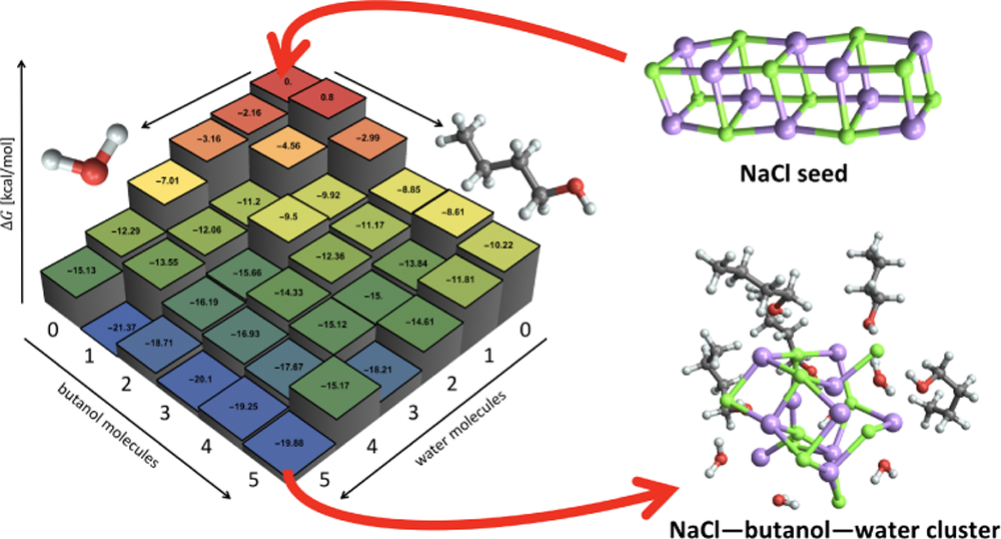

Using a combination of quantum chemistry and cluster size distribution dynamics, we study the heterogeneous nucleation of *n*-butanol and water onto sodium chloride (NaCl)_10_ seeds at different butanol saturation ratios and relative humidities. We also investigate how the heterogeneous nucleation of butanol is affected by the seed size through comparing (NaCl)_5_, (NaCl)_10_, and (NaCl)_25_ seeds and by seed electrical charge through comparing (Na_10_Cl_9_)^+^, (NaCl)_10_, and (Na_9_Cl_10_)^−^ seeds. Butanol is a common working fluid for condensation particle counters used in atmospheric aerosol studies, and NaCl seeds are frequently used for calibration purposes and as model systems, for example, sea spray aerosol. In general, our simulations reproduce the experimentally observed trends for the NaCl–BuOH–H_2_O system, such as the increase of nucleation rate with relative humidity and with temperature (at constant supersaturation of butanol). Our results also provide molecular-level insights into the vapor–seed interactions driving the first steps of the heterogeneous nucleation process. The main purpose of this work is to show that theoretical studies can provide molecular understanding of initial steps of heterogeneous nucleation and that it is possible to find cost-effective yet accurate-enough combinations of methods for configurational sampling and energy evaluation to successfully model heterogeneous nucleation of multicomponent systems. In the future, we anticipate that such simulations can also be extended to chemically more complex seeds.

## 1 Introduction

Gas-to-liquid phase transitions are the central processes in atmospheric new particle formation,^1^ as well as in the operation of condensation particle counters (CPCs) used to study atmospheric aerosol. CPCs are based on growing particles to optically detectable sizes through condensation of a supersaturated working fluid vapor.^2^ Nucleation, in this context, the formation of the first embryos of the condensed phase within supersaturated vapor, can occur either homogeneously (without a pre-existing liquid or solid phase) or heterogeneously (condensation of gas molecules onto a surface of a pre-existing particle or droplet).^3^ The thermodynamics of the onset of nucleation on molecular level, that is, the first few adsorption steps, is not well described by models based on classical bulk thermodynamics. Many studies have computed first-principles thermodynamic data, for example, free energies, for the first steps of atmospherically relevant homogeneous nucleation processes. However, computational limitations prevent the application of similar purely first-principles approaches to heterogeneous nucleation due to the large size of the realistic seed particles.

Butanol (specifically, 1-butanol, also known as *n*-butanol) is the most common working fluid used in CPC-based measurements of atmospheric aerosol.^4^ During such measurements, a significant amount of water vapor may enter the particle counter from the ambient atmosphere, and this may affect the measurement efficiency. For instance, it has been reported that for particles with diameter below 3.5 nm, the relative humidity (RH) of the particle flow affects the diameter of the smallest particle that can be detected.^5^ In general, the efficiency of particle counting depends on the composition of both the particles and the working fluid. For example, when water is used as a working fluid, hygroscopic particles may have a lower detection limit than hydrophobic particles due to stronger interactions between the vapor molecules and the seed.^4^

Heterogeneous nucleation can also be affected by the electrical charge of the seeds. In ion-induced nucleation, condensation takes place on a molecular ion or a pre-existing charged particle. The energy barrier of ion-induced nucleation is usually lower than that of nucleation on neutral seeds of similar size because electrostatic forces enhance the interaction between charged seeds and condensing molecules.^3^ This has recently been demonstrated to be the case for condensation of butanol onto various monatomic ions.^6^

NaCl (salt) nanoparticles are a common type of atmospheric aerosol, as they are produced by sea spray.^7^ They are often used as a test aerosol for calibration of CPCs.^8^ However, NaCl particles are water soluble and may also be electrically charged.^9^ To conduct reliable measurements of such particles, it is essential to know how humidity, as well as seed charge, affects the nucleation process.

To the best of our knowledge, no study has modeled heterogeneous nucleation of butanol on NaCl with the aim of understanding condensation in CPCs. However, a few experimental findings on this and related systems have been reported. Tauber et al.^10^ showed that the activation of the NaCl seed is enhanced when the RH is increased, or when the neutral seeds become negatively charged. This is in line with earlier experimental studies on heterogeneous nucleation of a mixture of *n*-propanol and water vapors, which showed that the nucleation barrier decreases with the introduction of water, in agreement with binary heterogeneous nucleation theory predictions.^11−13^ Also, Winkler et al.^14^ showed that heterogeneous nucleation of *n*-propanol on WO_*x*_ was higher for negatively charged seeds than for neutral seeds. In this work, we use computational methods to model the first steps of heterogeneous nucleation of butanol on NaCl seeds and to evaluate the impacts of humidity, temperature, and seed charge.

## 2 Computational Methodology

### 2.1 Modeling Nucleation

Various modeling approaches are available for studying heterogeneous nucleation including AerCoDe,^15^ the Aerosol Dynamics, gas- and particle-phase chemistry model (ADCHAM),^16^ or the Atmospheric Cluster Dynamics Code (ACDC).^17^ ACDC (developed by McGrath et al.^17^) is a molecular-level approach based on solving the birth–death equations for a set of clusters. ACDC combined with quantum chemical formation free energies is a convenient tool for studying systems for which experimental kinetic data (collision and/or evaporation rates) are not available.

We explicitly simulate the first steps of heterogeneous nucleation, starting with a naked NaCl seed, which serves as condensation nuclei for the condensing vapors (butanol and water). Clusters grow by seed–vapor molecule collisions and shrink due to evaporation of individual vapor molecules.^18−20^ Although the smallest particles detected by the CPCs are much larger than 1 nm, we model the first steps of the growth process by simulating seed/vapor clusters up to ∼1 nm size, for which quantum chemical calculations are feasible. Our hypothesis is that the extent of nucleation is ultimately controlled by individual interactions between the seed and the vapor molecules, or between the adsorbed vapor molecules, and these interactions are likely to be similar throughout the process. Therefore, any difference observed in the adsorption energetics of the first few vapor molecules on the NaCl seeds should be reflected in the overall condensation trend, for example, for different seed or vapor types. Our simulations on a small cluster can thus provide at least qualitative insights into the effects of seed charge, seed size, and vapor type and the associated nucleation mechanisms. In particular, when the interaction between the first few vapor molecules and the seed is weak, the vapor molecules will rapidly evaporate, and the seed will not have the opportunity to grow. To simplify the modeling, we omit reactions involving pure vapor clusters (e.g., butanol or water dimers) as the concentrations of these clusters are at least 3 orders of magnitude lower than the considered vapor concentrations (calculated using the detailed balance equation for all studied conditions). Similarly, we do not consider collisions between seed molecules or fission reactions where the seeds break apart. However, we do account for possible structural changes of the seed due to vapor adsorption. As it is possible that full reorganization of the seeds may not occur on timescales of CPC measurements (on the order of seconds), the formation rates computed here should be interpreted as upper limits: lesser reorganization would lead to less stable clusters and thus higher evaporation and lower nucleation rates. The overall nucleation/particle formation rate in ACDC is defined as the rate at which clusters grow larger than some pre-determined limit. This formation rate depends on the collision and evaporation rates, the vapor concentrations or production rates, and the possible additional sink terms related, for example, to the instrumental setup (e.g., wall losses, dilution, or coagulation with background particles).^17,20^ In this work, such sink processes were not considered.

While modeling the adsorption of butanol and water on NaCl seeds with ACDC, we thus need to specify^3^

1 concentrations of the NaCl seeds, butanol, and water,
2 the list of cluster sizes and compositions involved in the nucleation process plus a definition of the “outgrowing” cluster types (which should ideally be large and stable enough that their evaporation rates are negligible compared to the collision rates at the simulated vapor concentrations),^3^
3 rates for all cluster–molecule collisions (in this work, these have been computed using the kinetic theory of gases^21^ for neutral seeds and the parameterization of Su and Chesnavich^22^ for electrically charged seeds), and
4 evaporation rates of all clusters (in this work, these have been estimated from quantum chemically computed formation free energies, together with the kinetic gas theory collision rates, using the detailed balance chemical equation^17^).

### 2.2 Studied System

In this work, we first examined homogeneous nucleation of butanol. Homogeneous nucleation is an undesirable phenomenon in CPC measurements, as it falsely increases the number of detected particles. CPCs are accordingly run using settings where homogeneous nucleation is minimal. Whether or not our modeling approach predicts homogeneous nucleation when run at the instrumental conditions is thus an important first sanity check for our simulation methods.

**Figure 1:**
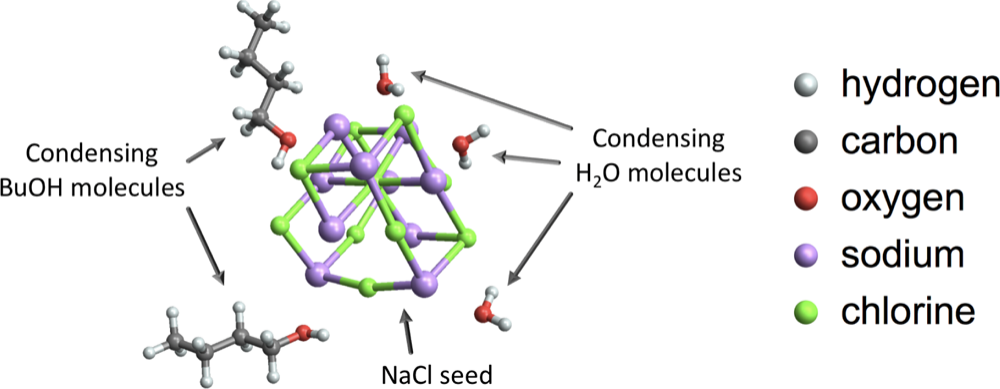
Example of the NaCl seed with condensing BuOH and H_2_O molecules.

Next, we examined how the heterogeneous nucleation of butanol (BuOH) on sodium chloride (NaCl) seeds depends on the modeled conditions: temperature, butanol saturation ratio, RH, seed size, and seed charge. Figure 1 shows an example of a studied molecular cluster consisting of a NaCl seed, two condensed butanol molecules, and three condensed water molecules, that is, the (NaCl)_10_(BuOH)_2_(H_2_O)_3_ cluster. We examined all (NaCl)_10_(BuOH)_0–5_(H_2_O)_0–5_ clusters, and we also studied small (NaCl)_5_ and large (NaCl)_25_ seeds as well as positive and negative seeds. Table 1 summarizes all the studied molecular systems.

**Table 1:** Composition of the Studied Clusters

study case	Na^+^	Cl^–^	BuOH	H_2_O
homogeneous nucleation	0	0	0–7	0
temperature	10	10	0–5	0
humidity	10	10	0–5	0–5
small seed	5	5	0–5	0
medium seed	10	10	0–5	0
big seed	25	25	0–5	0
neutral seed	10	10	0–5	0
positive seed	10	9	0–5	0
negative seed	9	10	0–5	0

### 2.3 Thermodynamic Properties of Clusters

A common assumption in cluster modeling studies is that the thermodynamic properties of each cluster type (in this context, each (NaCl)_*x*_(BuOH)_*y*_(H_2_O)_*z*_ cluster for any fixed set of *x*, *y*, and *z*) can be represented by the global Gibbs free energy minimum structure.^23,24^ Therefore, the first step in our modeling is configurational sampling: searching for the global minimum structure for each considered combination of *x*, *y*, and *z* listed in Table 1. We applied the approach described by Kubečka et al.^23^ First, the potential energy surface was explored using the artificial bee colony (ABC) algorithm^25^ implemented in the ABCluster program.^26,27^ The Na and Cl atoms, and the vapor (BuOH and H_2_O) monomer structures, were treated as rigid building blocks at this stage. The total number of building blocks in each simulation was thus 2*x* + *y* + *z*.

These building blocks were then used in the exploration (ABC) algorithm, where newly found structures were optimized using force-field (FF) methods. The FF included a combination of Lennard-Jones (LJ) and Coulomb interactions, where the LJ potential terms were taken from the CHARMM database.^28,29^ We assumed charges of +1 for sodium and −1 for chloride ions, respectively, while atomic partial charges for the butanol and water monomers were calculated using the MP2/6-31++G(d,p)^30−33^ method with NBO^34^ population analysis.

Four different conformers of BuOH were used as the initial rigid building blocks (see the Supporting Information for details).^35^ In the next step, 3000 lowest lying structures from ABCluster were optimized at the semi-empirical GFN2-*x*TB level with the XTB program.^36,37^ Based on the energy, dipole moment, and gyration radius of the optimized structures, we filtered out identical or energetically high-lying structures as redundant. After that, we selected a representative set of structures for vibrational frequency analysis, which was performed at the same computational level (GFN2-*x*TB^36,37^) to obtain Gibbs free energies at temperature *T* = 298.15 K.

GFN2-*x*TB (henceforth called “low level of theory”) provides similar geometries as much higher level and more expensive quantum chemical methods (DLPNO/aug-cc-pVTZ//LC-ωHPBE/def2TZVP; see below) in the sense that re-optimization at a higher level does not significantly change the structure of a particular local minimum conformer. Although the lowest free energy structures predicted by the two methods are often different, the values of the formation free energy predicted for a particular system (seed plus some number of adsorbed vapor molecules) by the two methods were generally fairly similar (see the comparison of free energies for both methods in Section S6 of the Supporting Information). Therefore, to lower the computational cost, the seed size and seed charge effects were only studied at the GFN2-*x*TB level.

For the higher-level calculations, we selected the lowest-energy structures from the GFN2-*x*TB results and optimized them at a density functional theory (DFT) level using the Gaussian 16 rev. A.03 program.^38^ As PBE-based methods are usually successful in quantum chemical modeling of NaCl crystals,^39−42^ we selected the LC-ωHPBE^43−46^ and mPW3PBE^47^ functionals. Ideally, we would have preferred to use the optPBE-vdw functional, as it yields adsorption energies in good agreement with experimental results, but it is unfortunately not supported by the Gaussian program.^38^ Also, we considered the ωB97XD functional because it can give accurate thermochemistry results.^48−50^ Both LC-ωHPBE and ωB97XD are long-range-corrected (ωB97XD additionally has added corrections for atom–atom dispersion interactions), which are required for more accurate description of van der Waals (vdw) interactions during modeling of NaCl crystals.^51,52^ Along with these functionals, we applied the def2-TZV, def2-TZVP, and def2-TZVPD basis sets^53,54^ to evaluate the necessity of including polarization or diffuse functions in the basis set. As the def2-TZVPD basis set is not included in the Gaussian library, the basis set parameters were extracted from the Basis Set Exchange database.^55^ To select the optimal computational level, we assessed their performance in predicting NaCl and butanol properties, and Na^+^/Cl^–^ interaction with butanol. As reported in Tables S5–S8, the LC-ωHPBE functional outperformed mPW3PBE47 and ωB97XD in predicting the relative Gibbs free energy of the *n*-butanol conformers, the lattice energy of the NaCl crystal, and the distance between the Na^+^ and Cl^–^ ions and the oxygen atom of butanol in ion/TGt complexes. Also, the results suggested that the inclusion of polarization functions in the basis set (def2-TZVP) might increase the error of butanol conformers’ relative energies and enhance the accuracy of NaCl crystal and butanol/NaCl calculations. On the other hand, the addition of diffuse functions to the basis set (def2-TZVPD) decreased the accuracy of the relative Gibbs free energies of the butanol conformers (Table S5) and the NaCl lattice energy (Table S6), although it improved the prediction of ion/TGt interaction when combined with ωB97XD (see Table S7). Including diffuse functions in the basis set also significantly increased the computational cost (Table S6). In the case of Na–Cl bond distance in the (NaCl)_10_ crystal, all levels predicted a value ranging from 2.71 to 2.77 Å, which are all smaller than the experimental value of the Na–Cl distance in bulk salt crystals (2.82 Å). This is expected as interatomic distances in free NaCl clusters are generally shorter than those of bulk NaCl.^56^ Overall, Table S8 indicates LC-ωHPBE/def2-TZVP as the optimal computational level. We note that previous studies also suggest that def2-TZVP provides a good balance between accuracy and computational cost.^57^

Since the generally used harmonic approximation does not accurately describe low-frequency vibrations, we applied the quasi-harmonic correction using the GoodVibes program^58^ to obtain cluster Gibbs free energies *G*^DFT^. The electronic energy was then corrected by single-point calculation using the domain-based local pair natural orbital-coupled cluster method DLPNO-CCSD(T)^59−62^ with the aug-cc-pVTZ basis set.^63,64^ These calculations were performed with Orca 4.0.1.2.^65^ The final Gibbs free energy *G* value was obtained as
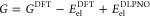
1where *E*_el_ refers to electronic energy.

For all systems, the reference temperature was set to 298.15 K (25 °C). At temperatures other than reference temperature, the Gibbs free energy was recalculated using the computed vibrational frequencies and moments of inertia. We assume here that the global minimum structures of the modeled clusters do not change with temperature over the studied temperature range. Even if this assumption was false, the associated error is very small, as the relative Gibbs free energies of different conformations of the same cluster do not vary significantly over the considered temperature range.^66^ See the Supporting Information for technical details of configurational sampling and quantum chemistry calculations.

### 2.4 Modeling Cluster Formation with ACDC

ACDC^17^ was used to model the time evolution of cluster distribution as clusters are formed through inelastic collisions.^17^ The birth–death equations describing cluster kinetics were numerically solved by MATLAB ode15s.^67^

**Figure 2:**
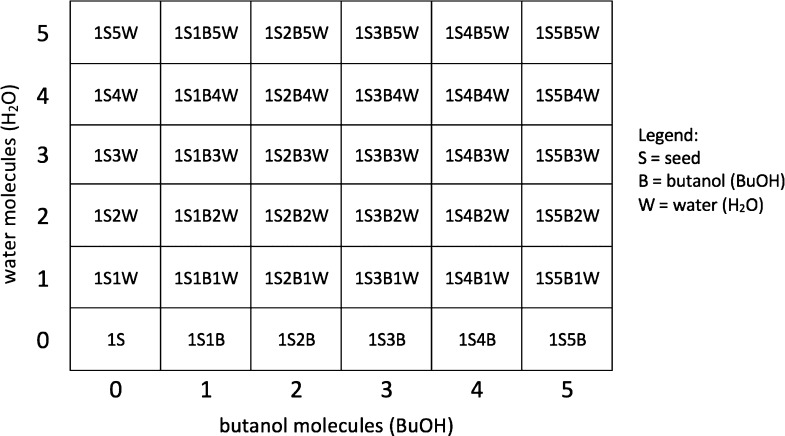
Diagram of the set of simulated clusters describing the seed–butanol–water nucleation process. For example, a cluster containing the NaCl seed, two butanol molecules, and three water molecules is denoted 1S2B3W.

The most extensive simulated system consists of 1 NaCl seed (S), up to five butanol molecules (B, *x*-axis), and up to five water molecules (W, *y*-axis) (see Figure 2). For each simulation, we first checked that the (concentration-corrected; see below) free energy of the addition of the fourth and fifth butanol molecules is negative. While this does not conclusively prove that our set of simulated clusters contains the critical cluster (as the system might contain local minima), it strongly suggests that the top of the nucleation barrier is within the simulated system.^3^

When a cluster grows out of the simulated system, we thus assume that it has nucleated (unless otherwise stated, see the Results and Discussion section). The rate of formation of these outgrowing clusters is defined as the nucleation rate *J*.

ACDC requires knowledge of the collision and evaporation rates for each species in the simulated system. The collision coefficients β_*ij*_ are calculated from kinetic gas theory for two spherical objects^17^
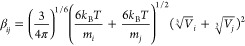
2where *m* is the mass, *V* is the volume, subscripts *i* and *j* refer to the cluster and the vapor molecule, respectively, *T* is the temperature, and *k*_B_ is the Boltzmann constant. We used the model of Su and Chesnavich^22^ for the calculation of electrically neutral molecule and charged cluster collision rate enhancement. Each collision of vapor and seed is assumed to lead to adsorption and immediate rearrangement of the entire cluster to the free energy global minimum configuration. Thus, we do not account for any unfavorable orientations of molecules during a collision or other steric barriers separating the structure from its global minimum. The evaporation rates can then be derived from the Gibbs free energies of formation of the clusters Δ*G*_*i*_^17^
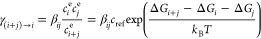
3where *c*_*j*_^e^ is the equilibrium concentration of species *j* and *c*_ref_ is the concentration corresponding to the reference pressure at which the free energies are calculated (here, 1 atm). As we consider only evaporation of vapor monomers in this study, *i* + *j* corresponds to the parent cluster, *i* to the daughter cluster, and *j* to a vapor molecule. Note also that in this case, Δ*G*_*j*_ = 0.

The standard Gibbs free energy of formation Δ*G*_*i*_^ref^ of a molecular cluster is calculated at 1 atm reference pressure *p*_ref_ and room temperature (25 °C) from the Gibbs free energies *G*_*i*_ of the individual species obtained from quantum chemistry calculations as described above
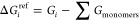
4The standard Gibbs free energies of formation can be converted to the Gibbs free energies of formation in the nucleating system as defined in classical nucleation theory when we know the monomer partial pressures *p*_*i*_
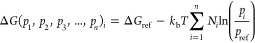
5where *n* is the number of components in the cluster and *N*_*i*_ is the number of molecules of type *i* in the cluster.

As mentioned before, we did not account for additional losses such as dilution, wall losses, or coagulation in this general study, as they usually differ between instrumental setups. For instance, most of the losses come from dilution (30–90%). This type of losses can be quantified from the flow rate. All CPC instruments also have some size-dependent losses (<90%), as indicated, for example, by the Gormley and Kennedy equation (for greater detail, see ref (8)).

We set the sources of the seeds, butanol monomers, and water monomers such that their concentrations remained constant during each simulation. For the seed, we used a typical experimental concentration of 10^4^ cm^–3^ for all simulations.^14^ The temperature, the butanol saturation ratio, and the humidity (water monomer concentration) were also kept constant within each simulation but were varied between different simulation runs to test their effect on the nucleation rate. The butanol saturation ratio was calculated as the ratio of the actual butanol vapor pressure and the saturation vapor pressure at a given temperature.^68^ Further, we also studied the effects of seed size and seed charge.

Finally, we simulated each system until it reached a steady-state condition, at which the concentrations of all species and the nucleation rate did not change over time. We report the results as ratios of the nucleation rate *J* compared to the nucleation rate at some reference condition *J*_ref_ (*J*/*J*_ref_).

## 3 Results and Discussion

### 3.1 Homogeneous Nucleation of Butanol

Typically, butanol vaporizes at 40 °C in CPCs, and the resulting vapor supersaturates as it is subjected to a temperature drop. The typical experimental nucleation temperature in CPCs ranges between 10 and 25 °C, and the butanol saturation ratio ranges from 1 to 5 (the butanol saturation vapor pressure at 25 °C is 919.2 Pa).^10,69^

**Figure 3:**
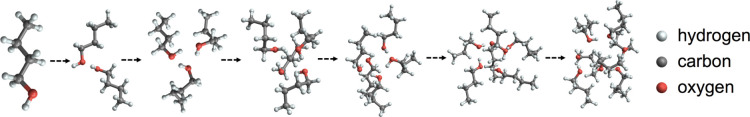
Global minimum structures of the butanol clusters at *T* = 25 °C and the DLPNO-CCSD(T)/aug-cc-pVTZ//LC-ωHPBE/def2TZVP level of theory.

We performed configurational sampling of the (BuOH)_1–7_ butanol clusters as described in the Computational Methodology section and computed their formation free energies. Figure 3 shows the global minimum structures of these clusters at 25 °C. The figure clearly shows that the hydrophilic hydroxyl groups of the butanol molecules point toward each other, while the hydrophobic alkyl chains point out of the clusters. Intermolecular hydrogen bonds are in this case the main drivers of cluster formation.

**Figure 4:**
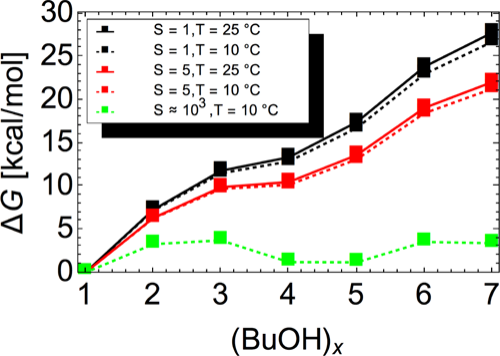
Gibbs free energy profiles of pure butanol cluster formation at different conditions, at the DLPNO-CCSD(T)/aug-cc-pVTZ//LC-ωHPBE/def2TZVP level of theory. The free energies are computed using the actual butanol monomer concentration, see eq 5. The green line illustrates how large the saturation ratio would need to be for the critical cluster to lie within the simulated set of clusters.

Figure 4 shows that the Gibbs free energy profiles for pure butanol cluster formation increase steadily as a function of cluster size and do not exhibit a maximum for any CPC temperature or butanol saturation ratio. This indicates that the critical cluster size is larger than seven molecules. Moreover, the graph implies that the nucleation barriers are several times greater than 1 *RT* (*RT* = 0.593 kcal/mol at 25 °C or *RT* = 0.563 kcal/mol at 10 °C). Additionally, the figure shows that for the critical cluster to be found within the simulated set of clusters, a saturation ratio ≈3 orders of magnitude greater is required (see the green line, where (BuOH)_3_ represents the critical cluster). Consequently, we can safely neglect the homogeneous nucleation of butanol in our simulations, which is in line with the observed behavior of CPCs at these conditions.

### 3.2 Heterogeneous Nucleation

#### 3.2.1 Butanol Clustering onto a NaCl Seed

**Figure 5:**
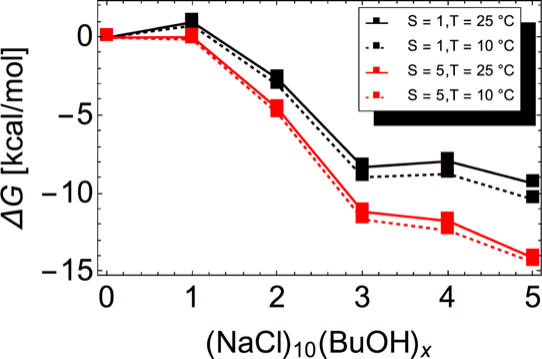
Gibbs free energy profiles for butanol clustering onto a (NaCl)_10_ seed at the DLPNO-CCSD(T)/aug-cc-pVTZ//LC-ωHPBE/def2TZVP level of theory. The free energies are computed using the actual butanol monomer concentration, see eq 5.

Figure 5 shows the Gibbs free energy profiles for butanol clustering onto a (NaCl)_10_ seed in several different conditions. Adsorption of the first and fourth butanol molecules are associated with low barriers at *S* = 1. The latter barrier disappears at *S* = 5. All other adsorption steps are barrierless at all studied conditions. The corresponding evaporation rates of butanol are consequently low due to the substantial decreases in the Gibbs free energy, and nucleation is thus proceeding close to the kinetic limit.

**Figure 6:**
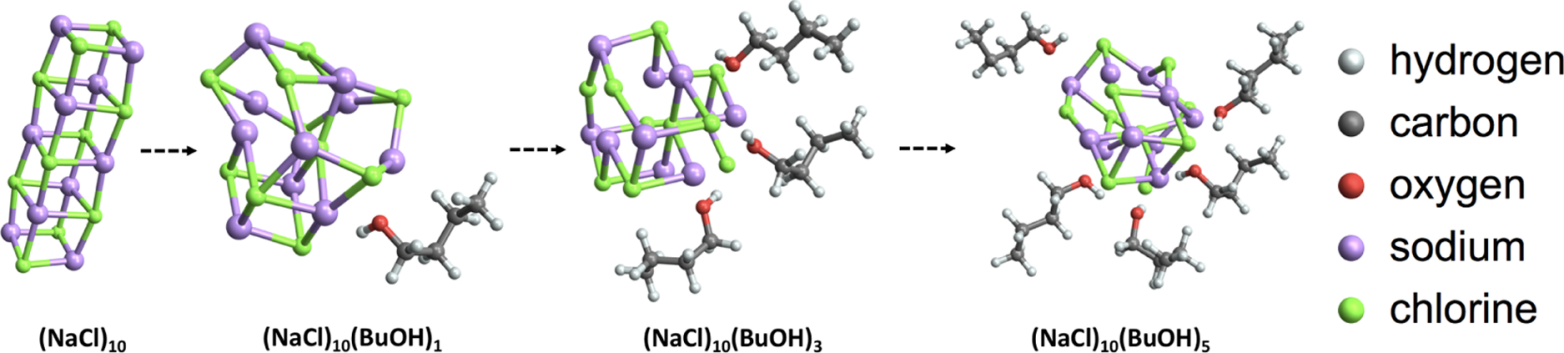
Illustrations of seed structural changes and butanol orientation during butanol clustering onto the NaCl seed.

Figure 6 shows a few examples of the global minimum structures with different numbers of butanol molecules added to the salt seed. The NaCl seed undergoes structural changes as the number of butanol molecules increases, but it remains compact in the center of the growing cluster. Here, we caution that our modeling assumes that clusters are fully reorganized to their global free energy minimum structures in between each collision with vapor molecules. If cluster reorganization in reality is incomplete, this leads to an overprediction of the nucleation rates. However, as our focus here is on comparing relative nucleation rates (e.g., between different conditions), possible errors due to incomplete reorganization will at least partially be cancelled out. We note that cluster reorganization can happen both via thermal reactions, on the timescales of seconds, and with the aid of excess energy from vapor adsorption, on the timescale of nanoseconds. Doye and Wales^70^ showed that the transition barriers between the minima of the (NaCl)_35_Cl^–^ seed ranged from a few to tens of kcal/mol. The barriers of 10 kcal/mol can easily be surmountable both by thermal reactions (assuming room temperature and a timescale of seconds) and by excess energy from condensation (see Table S8). In contrast, the barriers of several tens of kcal/mol are likely insurmountable. Thus, it seems likely that at least some reorganization barriers for (NaCl)_10_ can be overcome on the characteristic timescales of CPC instruments.

The adsorbed butanol molecules are oriented with their hydroxyl group toward the NaCl seed, leaving the alkyl chains pointing outward. The main stabilizing interactions between the seed and butanol molecules are Coulombic interactions between Na^+^ and the negatively charged hydroxyl oxygen from butanol and between Cl^–^ and the positively charged hydroxyl hydrogen of the butanol molecules. In addition, in the larger clusters, hydrogen bonds are also formed between the hydroxyl groups of several butanol molecules. Because of this hydrogen bonding, butanol preferably concentrates on one side of the seed at the beginning. However, as the nucleation process continues, butanol also adsorbs on the other sides of the seed.

**Figure 7:**
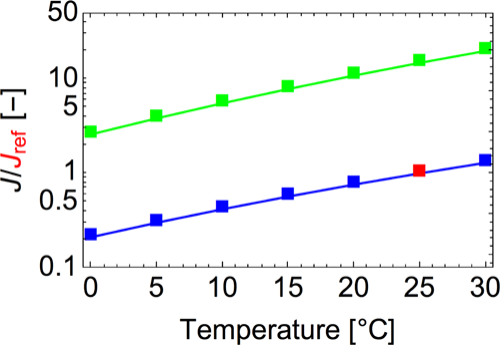
Heterogeneous nucleation rate as a function of temperature for the butanol saturation ratio of 1 (blue line) and ratio 5 (green line). The rates are plotted relative to that obtained at *S* = 1 and 25 °C (red point).

**Figure 8:**
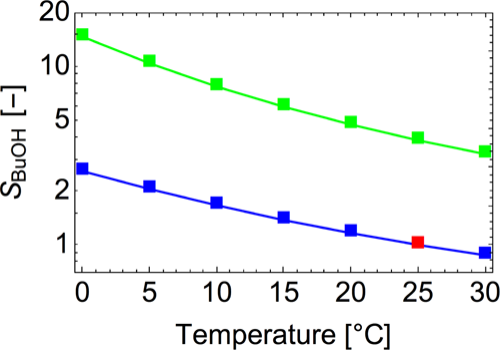
Butanol saturation ratio as a function of temperature for constant nucleation rates of *J*/*J*_ref_ = 1 (blue line) and *J*/*J*_ref_ = 10 (green line). *J*_ref_ corresponds to the nucleation rate at *S* = 1 and *T* = 25 °C (red point).

Figure 7 shows the nucleation rate predicted by ACDC as a function of temperature at saturation ratios of 1 and 5. As described above, the nucleation rate is calculated as the rate of formation of the outgrowing clusters, that is, the (NaCl)_10_(BuOH)_6_ cluster and the larger cluster. The rates are plotted relative to that obtained at *S* = 1 and 25 °C. Note that when the temperature increases, the equilibrium concentration of vapors corresponding to a certain saturation ratio also increases strongly. If Figure 7 was plotted with constant vapor concentrations rather than constant supersaturations, the nucleation rates would decrease as a function of temperature as the cluster evaporation rates increase. At a constant saturation ratio, the nucleation rate is enhanced when the temperature increases, which is a general rule, also known as the second nucleation theorem,^71^ that follows from statistical mechanics. One way to rationalize this is to start from the fact that the heterogeneous nucleation process is more favorable (has a lower barrier) than the corresponding homogeneous clustering. As seen from, for example, the Arrhenius equation, higher barriers almost inevitably imply higher temperature sensitivities and vice versa. Therefore, the temperature sensitivity of the heterogeneous nucleation rate, which depends on how strongly vapor molecules interact with the seed, is generally weaker than that of the saturation vapor pressure (which is a measure of how strongly the vapor molecules interact with each other). The results in Figure 7 also indicate that the butanol onset saturation ratio is decreasing with the increase of temperature, in agreement with the Kelvin prediction. This is illustrated in detail in Figure 8.

The experiments performed by Tauber et al.^10^ imply that the onset saturation ratio of butanol slightly decreases with temperature for seeds smaller than 3.5 nm, in agreement with the Kelvin prediction^72^ and with our results (as the diameter of the seed we are modeling is about 0.67 nm). Both experimental and computational results also agree that the temperature dependence of the onset saturation ratio is quite weak. However, Tauber et al.^10^ show that the trend changes, and the onset saturation ratio increases with temperature, when the initial seed is larger than 3.5 nm. This change in trend may be related to the onset of dissolution of the NaCl surface, which may become more favorable as the seed curvature decreases. Schobesberger et al.^73^ use *n*-propanol instead of *n*-butanol but show similar results as Tauber et al. As shown in Figure 6, the interaction of butanol with the seed is not strong enough to dissolve the NaCl ions when the seed is small: even though the seeds undergo structural changes, they still remain compact within the cluster.

#### 3.2.2 Humidity Effect

**Figure 9:**
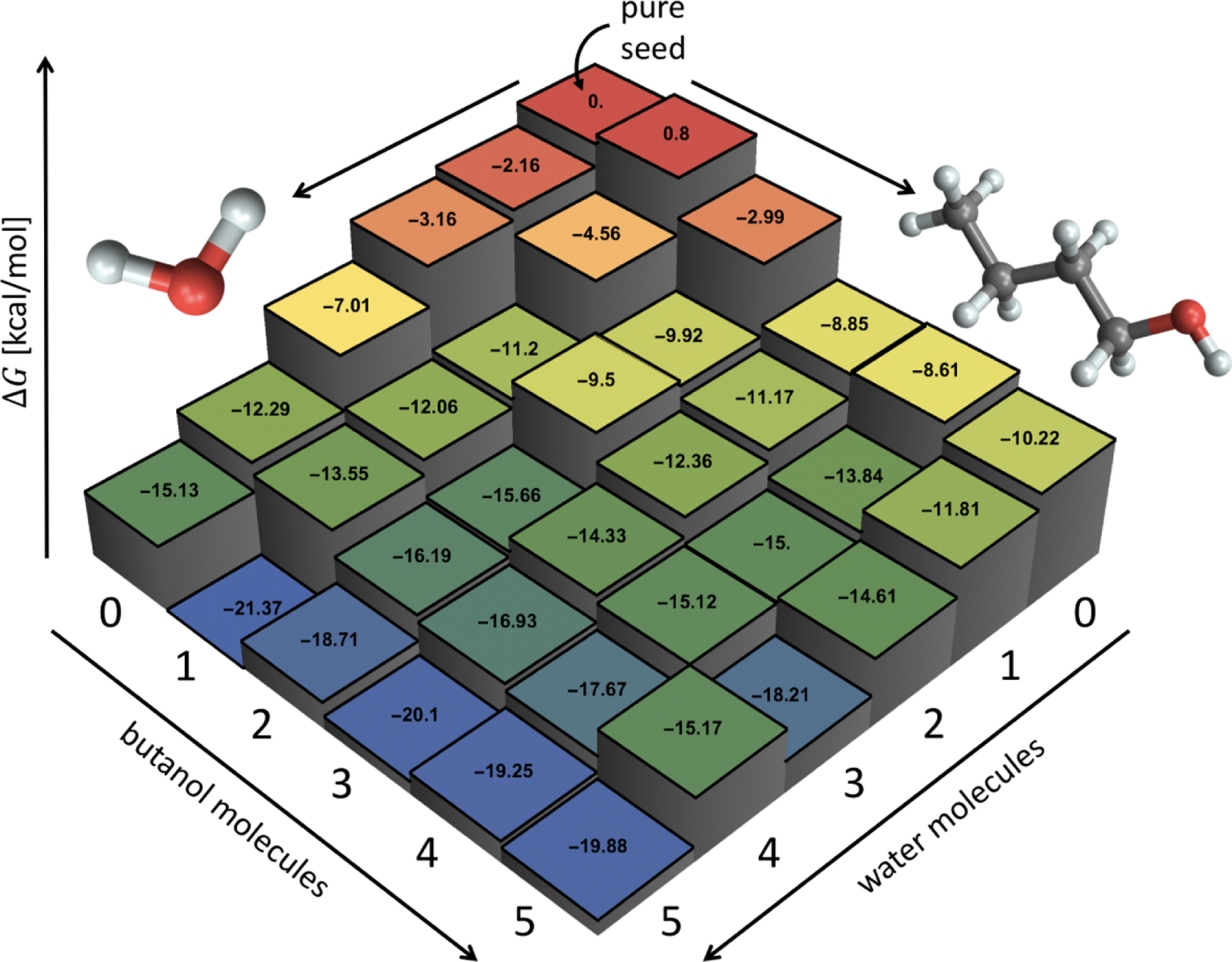
Example of Gibbs free energies of formation at *T* = 25 °C, butanol saturation ratio of *S* = 1, and humidity of RH = 10% at the DLPNO-CCSD(T)/aug-cc-pVTZ//LC-ωHPBE/def2TZVP level of theory. Each box corresponds to one seed–butanol–water clusters with the given numbers of water and butanol molecules attached to the seed. The free energies have been computed using the actual vapor concentrations.

To evaluate the effect of humidity on the nucleation process, we performed configurational sampling of the (NaCl)_10_(BuOH)_0–5_(H_2_O)_0–5_ seed–butanol–water clusters as described in previous subsections. Figure 9 shows the Gibbs free energy profiles of the nucleation process in the presence of water at 25 °C, a butanol saturation ratio of 1, and 10% humidity. This corresponds to a water concentration of [H_2_O] = 7.70 × 10^16^ cm^–3^ and the butanol concentration of [BuOH] = 6.94 × 10^16^ cm^–3^. The free energies have been computed using the actual vapor concentrations. A small barrier is visible in the seed–butanol direction, which can already be seen in Figure 5 (illustrating pure butanol condensation on the NaCl seed). Attachment of several water molecules to the seed is, in this case, barrierless. Here, we emphasize that we only study the first steps of seed activation. As the water supersaturation is far below 1, a barrier for (pure) water addition is very likely to exist when several condensation layers are formed. Even though pure water condensation is thus improbable, the presence of water clearly enhances the first steps of butanol condensation on the NaCl seed because it helps to stabilize the clusters. We note that for the water-containing systems, there are multiple ways of defining outgrowing stable clusters. Here, we have used a simple definition: if a sixth butanol molecule has been attached to the seed, the cluster is assumed to be stable. In contrast, if a sixth water molecule is attached to the seed, we assume that it immediately evaporates back. We have tested that the choice of outgrowing cluster definition does not qualitatively change our results. Additionally, in all ACDC simulations, the time-independent steady state is reached within less than 0.1 μs. This suggests that on the experimental timescale (100 ms–1 s), no seeds are trapped in any local free energy minima. For more details, see Section S8 in the Supporting Information.

**Figure 10:**
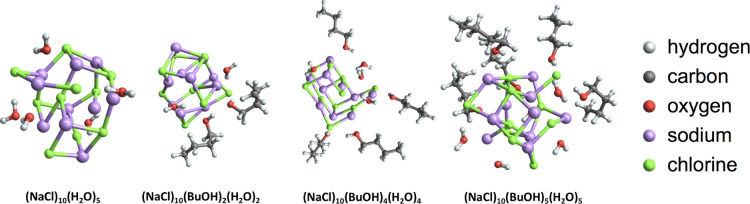
Structural changes of the seed as well as butanol and water orientation during butanol–water condensation on the NaCl seed.

Figure 10 shows several seed–butanol–water clusters. Comparing this figure to Figure 6, we can see that the seed undergoes larger structural changes in the presence of water. Some of the Na^+^ and Cl^–^ ions are pushed outward from the seed and are stabilized by water molecules. However, they are still connected to the seed core by at least one bond. This implies that either larger seeds or seeds surrounded by a greater number of water molecules would likely be able to dissolve into the liquid phase of the condensed molecules. The fact that our conformational sampling approach is able to capture the onset of the seed dissolution process indicates that similar tools (possibly without the computationally expensive final DFT or coupled cluster calculations) could be used to also study this dissolution in larger systems.

**Figure 11:**
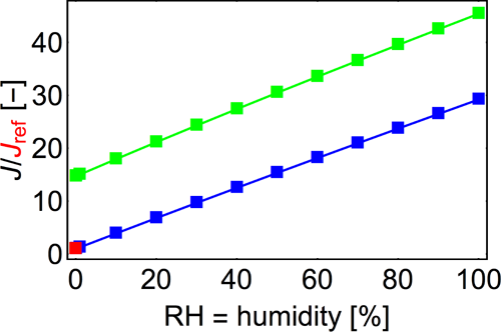
Heterogeneous nucleation rate as a function of RH for butanol saturation ratios of 1 (blue line) and 5 (green line). The nucleation rate is shown with respect to the rate at *T* = 25 °C, *S* = 1, and RH = 0% (red point).

The effect of humidity on the nucleation rate at *T* = 25 °C is shown in Figure 11. Humidity can enhance the nucleation rate by up to 1 order of magnitude. The water molecules both help stabilize the formed cluster (reducing evaporation rates) and increase their collision cross sections (increasing collision rates). Together with the seed restructuring, the presence of water may also increase the total polarity of cluster, which could further enhance the collision rate through dipole–dipole or ion–dipole interactions.^74^ However, this potential effect is not modeled in this study. It is also known that larger NaCl seeds may “shrink” due to dissolution in the presence of water^75^ and *n*-propanol.^11^ Tauber et al.^10^ reported that the degree of NaCl nanoparticle shrinkage in the presence of water vapor is directly proportional to particle size; that is, larger particles shrink more than the smaller ones. However, we note that this “shrinkage” refers only to the solid NaCl core of a NaCl–butanol–water cluster: the actual overall cluster size may not be affected by the process or could potentially even increase.

Our results agree with those of Tauber et al.,^10^ who found that humidity lowers the required onset saturation ratio of butanol on salt.

#### 3.2.3 Seed Size and Charge

To exhaustively explore the effects of seed size and charge on heterogeneous nucleation rates in the NaCl–BuOH–water system, a very large number of clusters would need to be studied. Because of the high computational cost associated with such an investigation, we have here significantly restricted the set of studied clusters by evaluating the effects of seed size and charge only for the NaCl–BuOH system, with no water molecules present. Furthermore, we have used a lower level of theory to estimate the relative stability of the studied clusters. Optimization of low-level structures at the high level of theory adjusts the bond lengths and atom–atom distances but did not change the overall bonding pattern or orientation of the molecules in the clusters studied here. However, the relative (free) energies predicted by the two methods often varied, leading to reordering with respect to free energies, that is, the global minimum at low level of theory was usually not the global minimum at the high level of theory. Nevertheless, despite this issue, the GFN2-*x*TB method seems to predict reasonably similar cluster structures and thermodynamics compared to the DLPNO-CCSD(T)/aug-cc-pVTZ//LC-ωHPBE/def2TZVP method used in previous subsections. (See the Supporting Information for comparison of the Gibbs free energy profiles of (NaCl)_10_(BuOH)_0–5_ calculated with these two methods.) Therefore, in this subsection, we performed configuration sampling and thermodynamic stability evaluation using GFN2-*x*TB as the highest level of theory.

**Figure 12:**
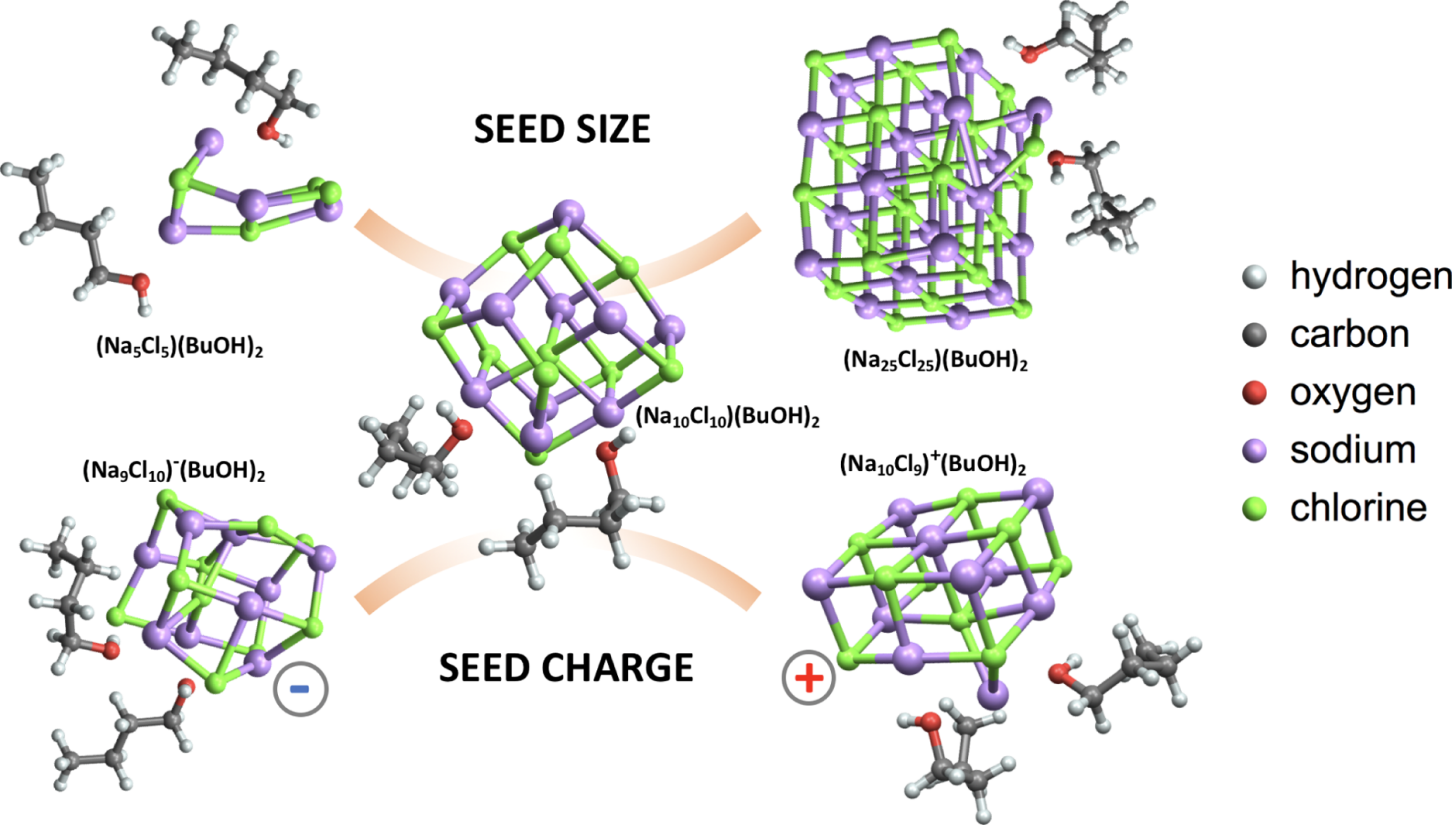
Seed size and charge variations during butanol condensation on different NaCl seeds.

Figure 12 gives an overview of the structural variations found for the different seeds studied in this section. As can be seen, changing the seed size does not alter the nature of the nucleation process described in the previous subsections. The main effects of the seed size on the initial step of heterogeneous nucleation are (1) greater initial collision cross sections of the larger seeds (see below) and (2) greater areas available for the first and subsequent solvation layers. The latter might affect the condensation processes for larger clusters but is not investigated in this study due to the limited number of vapor molecules included in our simulations. In the charged clusters, we observed two different types of seed–butanol interactions. In the negatively charged seeds, the butanol molecules mainly interact with Na^+^ ions through their hydroxyl hydrogen atoms. In contrast, in the positively charged seeds, the butanol molecules mainly interact with Cl^–^ ions through their hydroxyl oxygen atoms.

**Figure 13:**
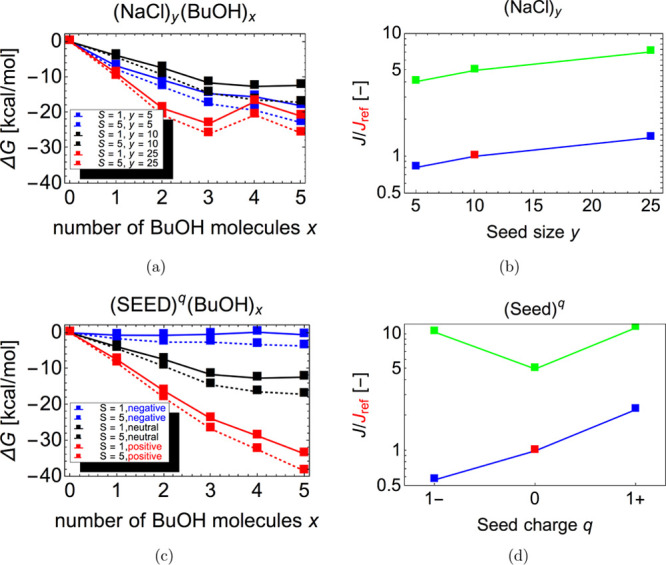
Effect of seed size and seed charge on the first steps of heterogeneous seed–butanol nucleation. In all figures, we used the GFN2-*x*TB level (low level of theory) and temperature *T* = 25 °C. The left figures show Gibbs free energies of formation computed using actual vapor concentrations. Full lines correspond to saturation ratios of 1 and dashed lines correspond to saturation ratios of 5. The right figures show the nucleation rates of butanol on NaCl seeds for butanol saturation ratios of 1 (blue) and 5 (green). The nucleation rates are shown with respect to the (NaCl)_10_ seed at *S* = 1 (red point). (a) Formation Gibbs free energy profiles with varied seed size (*y*). (b) Nucleation rates with varied seed size (*y*). (c) Formation Gibbs free energy profiles with varied seed size charge (*q*). (d) Nucleation rates with varied seed size (*q*).

Figure 13a shows the Gibbs free energy profiles of the (SEED) (BuOH)_0–5_ clusters with the seed sizes (diameters): (NaCl)_5_ (approx. 0.55 nm), (NaCl)_10_ (approx. 0.67 nm), and (NaCl)_25_ (approx. 0.77 nm). In conditions corresponding to CPCs (butanol saturation ratio of 1–5 and temperature of 25 °C), there is no nucleation barrier for any of the studied seed sizes. Somewhat surprisingly, the Gibbs free energy profile decreases faster for the (NaCl)_5_ seed than for the (NaCl)_10_ seed. This may be related to stronger Coulombic interactions in the smaller system due to a sharp corner in the seed geometry (see the Supporting Information). For the largest (NaCl)_25_ seed, the decrease in the Gibbs free energy profile is generally the strongest, as we would expect, for example, from classical thermodynamics. However, the adsorption of four butanol molecules is unexpectedly unfavorable, resulting in a low local maximum in the free energy profile. This may be an artifact caused by a failure of the low-level GFN2-*x*TB calculations or the configurational sampling for the global minimum structure of the (NaCl)_25_(BuOH)_4_ cluster. After excluding this anomaly, we can claim that regardless of the seed size, nucleation is generally thermodynamically favorable. Even though the evaporation rates are in some cases of the same magnitude as the collision rates, implying that clusters with a larger number of butanol molecules should in principle be included in the simulations, we believe that the results are sufficient for a qualitative comparison. Therefore, the increase of the nucleation rate with seed size (see Figure 13b) is mainly caused by the increase of the collision cross section with seed size. Larger seeds can also provide more suitable adsorption locations for butanol, as shown by Li and Hogan.^76^

Figure 13c shows the Gibbs free energy profile of the (SEED)^CHARGE^(BuOH)_0–5_ clusters with negative, (Na_9_Cl_10_)^−^, neutral (Na_10_Cl_10_), or positive (Na_10_Cl_9_)^+^ electrical charge. According to the Gibbs free energy profiles, nucleation is most favorable on the positively charged seed and the least favorable on the negatively charged seed. For the negatively charged seed, and especially for *S* = 1, the formation free energies at actual monomer concentrations are close to zero, indicating that the evaporation rates are close to the collision rates. Thus, the nucleation rates in the negatively charged system are well below the kinetic limit for low butanol saturation ratios (Figure 13d). However, for *S* = 5, the nucleation rate in the negative system exceeds that of the neutral system due to the charge enhancement of the collision rate (see Subsection 2.3—Modeling cluster formation with ACDC). Also, the nucleation rates in the negative and positive systems at *S* = 5 are almost identical, as the Su and Chesnavich method^22^ predicts identical collision rates for negative and positive clusters. Accounting for possible structural and orientational effects on collision rates would require explicit molecular dynamics simulations, which are outside the scope of this study.

Our results are in qualitative agreement with the experimental results of Li and Hogan,^76^ who reported that the adsorption of butanol vapor onto NaCl seeds for a butanol saturation ratio range of 0–0.435 (0–400 Pa) is charge state dependent, with a positive sign preference. However, in conflict with our results, the experimental nucleation study conducted by Tauber et al.^10^ found that negative charge enhanced seed activation for NaCl seeds below 3.5 nm in diameter, with positive charge having no effect in this size region. For larger seeds, they reported that charge does not affect nucleation. We note that the Tauber et al.^10^ study did not perform mass spectrometric characterization of their furnace-produced seeds, and the presence of impurities in the seeds of various size classes can thus not be ruled out. Based on the very large difference between the Gibbs free energy profiles illustrated in Figure 13c, we also suggest that the experimental observations might be related to higher collision rates (rather than lower evaporation rates) in the negatively charged systems due to mechanisms not accounted for in this study.

## 4 Conclusions

In this work, we studied the first steps of heterogeneous nucleation of butanol on NaCl and the effect of various variables including temperature, butanol saturation ratio, humidity, and seed size and charge. The simulations were performed using ACDC,^17^ and the thermodynamic stability of the clusters was evaluated through computational chemistry.

As a sanity check of our approach, we first tested that butanol does not homogeneously nucleate under typical CPC conditions: a temperature of 10–25 °C and a butanol saturation ratio of 1–5.^10,69^ In agreement with experiments, and with classical thermodynamic predictions, our modeling indicates negligible rates for homogeneous nucleation in these conditions.

Our simulations show that increasing either temperature or humidity enhances the rate of heterogeneous nucleation of butanol on NaCl seeds at constant butanol supersaturation. The addition of a small number of water molecules to the clusters is thermodynamically favorable even at low relative humidities. This adsorption of water both decreases the evaporation rate of butanol molecules and increases the cluster size and consequently the collision cross section. Water condensation also affects the seed structure and possibly the polarity.

Our results show that a combination of low-level conformational sampling with high-level quantum chemical energy evaluations is able to reproduce experimentally observed trends for heterogeneous nucleation in the NaCl–BuOH–H_2_O system, as well as provide insights into the molecular-level interactions, including at least the first steps of seed dissociation.

The effects of seed size and charge were studied at a low level of theory (GFN2-*x*TB), as this method was found to qualitatively reproduce structural and thermodynamic trends predicted by higher-level methods (DFT and coupled cluster). As expected, and in agreement with experimental results,^10,76^ we found that larger seeds lead to higher heterogeneous nucleation rates, mainly due to higher collision rates. Our simulations of charged clusters demonstrate that the molecular-level seed–vapor interactions are quite different for positively and negatively charged clusters. We also predict that the positively charged clusters are considerably more stable in agreement with experimental results for sub-saturated butanol adsorption but in apparent disagreement with the results on heterogeneous nucleation.

## Supporting Information Available

Butanol conformer structures, technical details of computational methods, method validation, formation free energies of global minima, nucleation rates corresponding to all related figures, comparison of low and high level of theory, and seed partial charges (PDF)
Geometry and vibrational frequencies (quantum chemistry output files) of all energetically lowest-lying seed–butanol–water clusters (ZIP)
