# Supplementary material for: Heterogeneous Nucleation of Butanol on NaCl: A Computational Study of Temperature, Humidity, Seed Charge, and Seed Size Effects
Source: J Phys Chem A. 2021 Mar 31;125(14):3025–36. doi: 10.1021/acs.jpca.0c10972 (PMC8054243; doi:10.1021/acs.jpca.0c10972)
Supplement: Supplementary file 1 — jp0c10972_si_001.pdf [file jp0c10972_si_001.pdf]

Supporting Information:

Heterogeneous Nucleation of Butanol on NaCl: Computational Study of Temperature, Humidity, Seed Charge, and Seed Size Effects

Antti Toropainen,[†,§] Juha Kangasluoma,[†] Theo Kurtén,[‡] Hanna Vehkamäki,[†]
Fatemeh Keshavarz,[†,¶] Jakub Kubečka*,[†,§]

† University of Helsinki, Institute for Atmospheric and Earth System Research/Physics, Faculty of Science, P.O. Box 64, Helsinki, FI 00014

‡ University of Helsinki, Department of Chemistry, Faculty of Science, P.O. Box 64, Helsinki, FI 00014

¶ LUT University, Department of Physics, School of Engineering Science, Lappeenranta, FI 53851

§ Contributed equally to this work

✆ +420 724946622

E-mail: jakub.kubecka@helsinki.fi

Table of Contents

S1. Initial butanol conformers S2

S2. Technical details of configurational sampling S6

S3. Validation of computational methods S7

S4. Formation free energy values S8

S5. Nucleation rate related tables S10

S6. Comparing low and high levels of theory S12

S7. Seed partial charges S13

S8. Evolution of cluster concentrations S14

References S15

S1. Initial butanol conformers

In the following subsections, we present the four configurations of butanol used in the initial steps of configurational sampling.[1]

Structure TGt

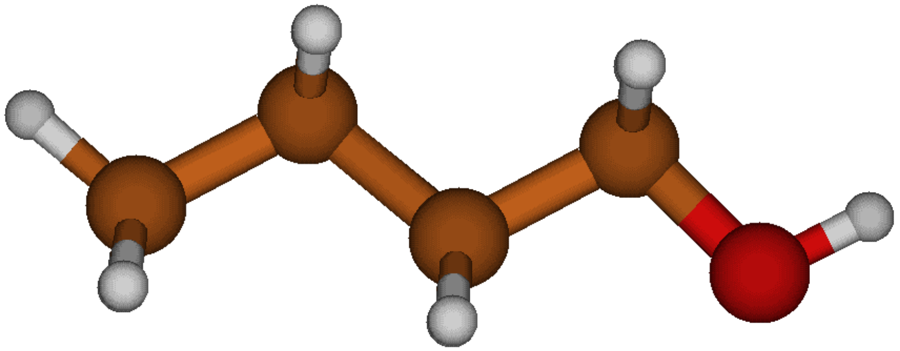

Figure S1. 3D model of TGt *n*-Butanol structure.

Table S1. Cartesian coordinates of the TGt *n*-butanol structure optimized with the second-order Møller-Plesset perturbation theory (MP2) and the 6-311++G(d,p) basis set.[1]

	Coordinate (Å)		
**Atom**	**x**	**y**	**z**
O	-2.41317	-0.42280	-0.00001
H	-3.23030	0.08155	0.00004
C	-1.31147	0.47430	0.00000
H	-1.33941	1.12175	0.88960
H	-1.33941	1.12175	-0.88959
C	-0.03207	-0.34217	0.00000
H	-0.03459	-0.99595	0.88075
H	-0.03459	-0.99595	-0.88074
C	1.22357	0.52971	0.00000
H	1.21215	1.18768	0.87895
H	1.21215	1.18767	-0.87896
C	2.50995	-0.29447	0.00000
H	2.56180	-0.93905	-0.88415
H	3.39585	0.34782	0.00000
H	2.56180	-0.93904	0.88416

Structure GGg

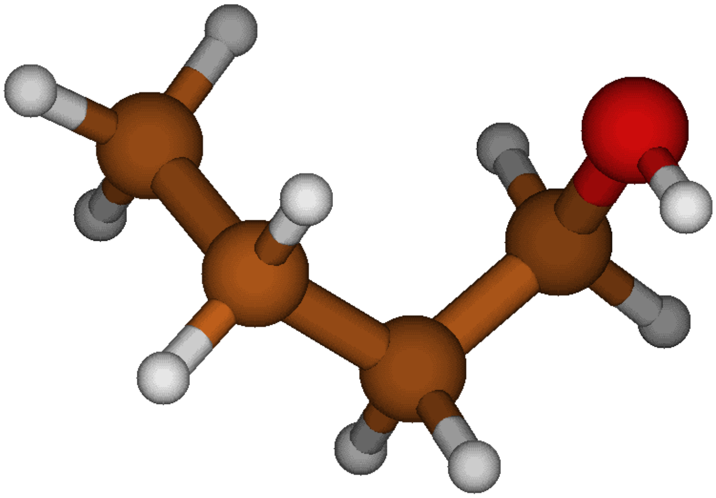

Figure S2. 3D model of GGg *n*-butanol structure.

Table S2. Cartesian coordinates of the GGg *n*-butanol structure optimized with the second-order Møller-Plesset perturbation theory (MP2) and the 6-311++G(d,p) basis set.[1]

	Coordinate (Å)		
**Atom**	**x**	**y**	**z**
O	-1.72285	-0.74594	0.39402
H	-2.10833	-0.18190	1.06654
C	-1.16405	0.07583	-0.61330
H	-1.94529	0.66179	-1.11353
H	-0.75459	-0.61080	-1.35571
C	-0.08153	1.01200	-0.09440
H	0.29501	1.59594	-0.94143
H	-0.54003	1.73226	0.59306
C	1.07970	0.31286	0.60951
H	0.69539	-0.23870	1.47076
H	1.75431	1.07668	1.00497
C	1.86289	-0.63708	-0.28841
H	2.22011	-0.12612	-1.18641
H	2.73218	-1.03928	0.23388
H	1.25203	-1.48406	-0.60473

Structure GG'g

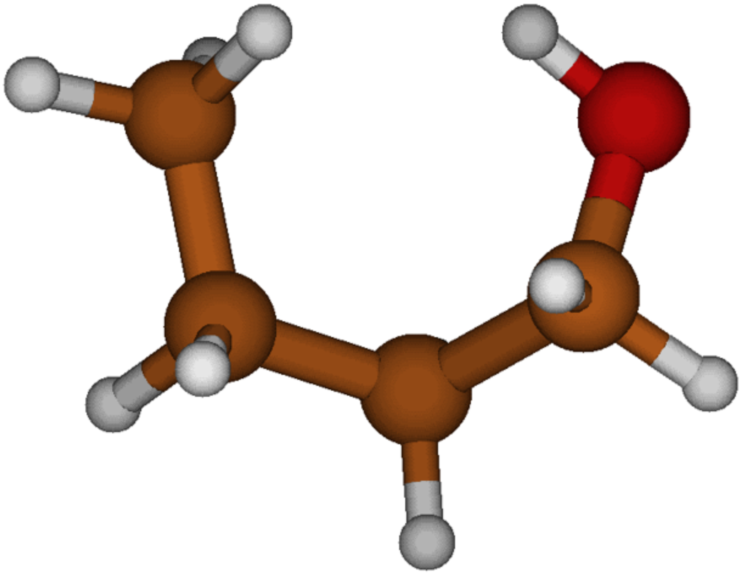

Figure S1. 3D model of GG'g *n*-butanol structure.

Table S1. Cartesian coordinates of the GG'g *n*-butanol structure optimized with the second-order Møller-Plesset perturbation theory (MP2) and the 6-311++G(d,p) basis set.[1]

	Coordinate (Å)		
**Atom**	**x**	**y**	**z**
O	-1.62664	-0.89911	-0.18316
H	-0.89203	-1.50199	-0.06667
C	-1.27364	0.36427	0.34896
H	-1.08075	0.29207	1.42882
H	-2.16375	0.98151	0.22204
C	-0.08839	1.02637	-0.34716
H	-0.19010	0.84972	-1.42237
H	-0.16748	2.10744	-0.19931
C	1.29301	0.58085	0.13291
H	1.41887	0.87816	1.17878
H	2.04746	1.13441	-0.43208
C	1.57899	-0.91238	0.00361
H	1.36972	-1.27160	-1.00701
H	2.62508	-1.13016	0.22349
H	0.98629	-1.50126	0.70970

Structure GGt

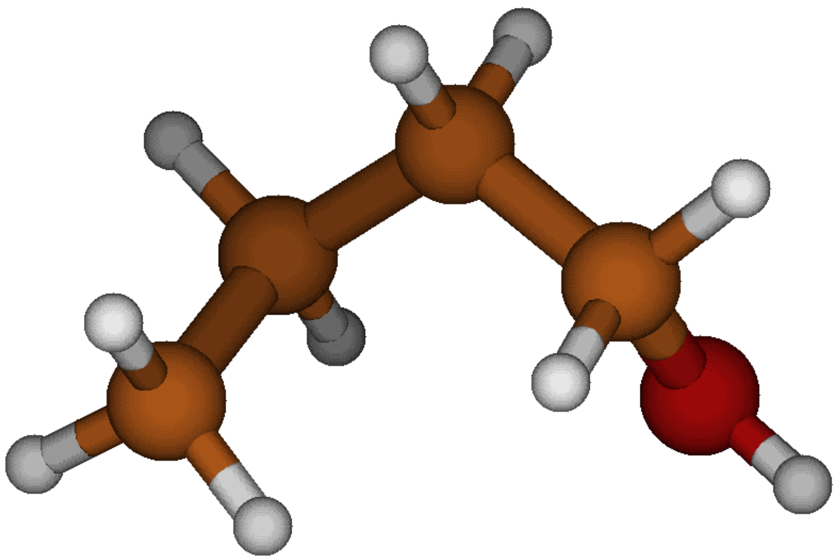

Figure S2. 3D model of GGt *n*-butanol structure.

Table S2. Cartesian coordinates of the GGt *n*-butanol structure optimized with the second-order Møller-Plesset perturbation theory (MP2) and the 6-311++G(d,p) basis set.[1]

	Coordinate (Å)		
**Atom**	**x**	**y**	**z**
O	1.68609	-0.63084	-0.49338
H	2.36004	-1.22500	-0.16393
C	1.16504	0.11870	0.58989
H	1.95087	0.72895	1.05224
H	0.76704	-0.54866	1.36490
C	0.06883	1.02754	0.06918
H	-0.30392	1.61891	0.91211
H	0.51332	1.73051	-0.64033
C	-1.09286	0.29813	-0.60346
H	-0.71516	-0.24514	-1.47185
H	-1.79246	1.04704	-0.98388
C	-1.83657	-0.66394	0.31500
H	-2.18610	-0.15797	1.21900
H	-2.70766	-1.08775	-0.18706
H	-1.20135	-1.49678	0.62220

S2. Technical details of configurational sampling

Summary of the individual steps with technical details:

1. **Potential energy surface exploration.**

In application of the artificial bee colony algorithm, the population was set to 20, the number of scout bees was set to 4, and the number of generations was set to 20. The small exploration efficiency is compensated by a great number of possible monomer combinations. Notably, 4 butanol conformers were included in configurational space exploration obtained from Moc *et al.*[1] The number of saved local minima over all combinations was set to 3000.

2. **Low-level optimization.**

The obtained local minimum structures were semi-empirically optimized using the GFN2-*x*TB method [2,3] with the very tight optimization criterion.

3. **Filtering and sampling**.

The redundant structures with relative energies higher than 15 kcal/mol were filtered out. Moreover, to minimize the computational cost, just 100 structures were uniformly selected (as different as possible) based on their gyration radius, energy and dipole moment.[4]

4. **Low-level thermodynamics.**

For the selected structures we performed vibrational frequency analysis using the GFN2-*x*TB method [2,3] to obtain Gibbs free energies at room temperature (T = 298.15 K = 25.15 °C).

5. **Sorting and selection.**

All 100 structures were sorted with respect to their Gibbs free energy and only the 20 lowest energy structures were selected for high-level quantum chemistry calculations. We assume that the lowest Gibbs free energy structures at the high level of theory are also the lowest (or close to the lowest) at the low level of theory.

6. **High-level thermodynamics.**

The selected structures were first optimized with loose optimization criterion at the LC-ωHPBE/6-31+G(d,p) level. This was done to save computational cost. The loosely re-optimized structures were then reoptimized with tight criteria using the LC-ωHPBE functional and the def2TZVP basis set (using the Gaussian program [5]). Cluster Gibbs free energies were obtained using the quasi-harmonic approximation using the GoodVibes program.[6] Finally, the electronic energy correction was done using the DLPNO-CCSD(T) method with the aug-cc-pVTZ basis set (using the ORCA program [7]). The high level of theory thus refers to the DLPNO-CCSD(T)/aug-cc-pVTZ//LC-ωHPBE/def2TZVP approach.

S3. Validation of computational methods

Table S5. Gibbs free energies of the *n*-butanol conformers relative to the most stable conformer in kJ mol^{-1}, at 298.15 K and 1 atm. The mean absolute error (MAE) values are calculated based on the CCSD(T)/aug-cc-pVTZ//CCSD/cc-pVDZ results.

Computational level	GGg	GG'g	GGt	TGt	MAE (%)
CCSD(T)/aug-cc-pVTZ//CCSD/cc-pVDZ	2.8	8.8	2.1	0.0	-
LC-ωHPBE/def2-TZV	2.4	9.0	2.6	0.0	13.56
LC-ωHPBE/def2-TZVP	2.8	10.0	2.8	0.0	15.92
LC-ωHPBE/def2-TZVPD	2.9	10.4	2.8	0.0	19.28
mPW3PBE/def2-TZV	2.6	8.9	3.0	0.0	16.93
mPW3PBE/def2-TZVP	3.0	9.5	3.1	0.0	21.18
mPW3PBE/def2-TZVPD	3.3	10.0	3.2	0.0	28.55
ωB97XD/def2-TZV	1.3	6.7	1.6	0.0	34.59
ωB97XD/def2-TZVP	1.5	7.5	1.7	0.0	25.73
ωB97XD/def2-TZVPD	1.4	7.6	1.6	0.0	28.47

Table S6. Na–Cl bond distance and lattice energies for the $(NaCl)_{10}$ crystals optimized at the evaluated computational levels. The lattice energies include zero-point energy correction. The lattice energy values in parentheses are absolute errors (%) relative to the experimental energy.

Level	NaCl distance (Å) [a]	Lattice energy (eV)	Total CPU time (min) [b]
Experimental value [8]	2.82	8.2	-
LC-ωHPBE/def2-TZV	2.72	7.5 (8.32)	68
LC-ωHPBE/def2-TZVP	2.71	7.7 (5.67)	1287
LC-ωHPBE/def2-TZVPD	2.71	7.4 (9.18)	6302
mPW3PBE/def2-TZV	2.74	7.5 (8.67)	51
mPW3PBE/def2-TZVP	2.73	7.7 (6.20)	912
mPW3PBE/def2-TZVPD	2.73	7.4 (10.11)	4916
ωB97XD/def2-TZV	2.77	7.4 (9.39)	83
ωB97XD/def2-TZVP	2.75	7.6 (6.93)	1272
ωB97XD/def2-TZVPD	2.75	7.3 (10.68)	6929

[a] The bond distances are the average of four Na–Cl bonds located in the middle of the $(NaCl)_{10}$ crystal. The experimental bond distance refers to bulk NaCl.
[b] Total CPU time accounts for both optimization and frequency calculation time.

Table S7. The distance between the Na^+ and Cl^- ions and the oxygen atom of butanol in ion/TGt complexes (TGt: the most stable butanol conformer) and the corresponding Gibbs free energies (G; kJ mol^{-1}) at 298.15 K and 1 atm. The values in parentheses are absolute errors (%) relative to the CCSD(T)/aug-cc-pVTZ//CCSD/cc-pVDZ results and mean absolute error (MAE) is the average of all errors.

Computational level	Na^+-O distance (Å)	Cl^--O distance (Å)	*G* (Na^+-TGt)	*G* (Cl^--TGt)	MEA (%)
CCSD(T)/aug-cc-pVTZ//CCSD/cc-pVDZ	2.22	3.04	-80.6	-49.0	
LC-ωHPBE/def2-TZV	2.18 (1.80)	3.07 (0.99)	-110.8 (37.43)	-56.3 (14.83)	13.76
LC-ωHPBE/def2-TZVP	2.21 (0.45)	3.03 (0.33)	-92.0 (14.16)	-56.2 (14.67)	7.40
LC-ωHPBE/def2-TZVPD	2.21 (0.45)	3.05 (0.33)	-91.6 (13.61)	-40.2 (18.02)	8.10
mPW3PBE/def2-TZV	2.17 (2.25)	3.06 (0.66)	-106.8 (32.55)	-61.0 (24.35)	14.95
mPW3PBE/def2-TZVP	2.20 (0.90)	3.02 (0.66)	-89.5 (11.00)	-61.0 (24.42)	9.24
mPW3PBE/def2-TZVPD	2.20 (0.90)	3.04 (0.00)	-87.8 (8.96)	-37.2 (24.08)	8.48
ωB97XD/def2-TZV	2.20 (0.90)	3.10 (1.97)	-110.1 (36.65)	-60.0 (22.23)	15.44
ωB97XD/def2-TZVP	2.24 (0.90)	3.06 (0.66)	-92.1 (14.29)	-60.4 (23.24)	9.77
ωB97XD/def2-TZVPD	2.24 (0.90)	3.08 (1.32)	-91.0 (12.96)	-44.5 (9.25)	6.11

Table S8. Overall error (%) of the evaluated computational levels against experimental values or CCSD(T)/aug-cc-pVTZ//CCSD/cc-pVDZ results in prediction of the n-butanol conformers' relative Gibbs free energies, NaCl lattice energy and TGt/ion (Na^+/Cl^-) binding.

Level	Conformers	NaCl crystal	TGt-ion binding	MAE (%)
LC-ωHPBE/def2-TZV	13.56	8.32	13.76	11.88
LC-ωHPBE/def2-TZVP	15.92	5.67	7.40	9.66
LC-ωHPBE/def2-TZVPD	19.28	9.18	8.10	12.19
mPW3PBE/def2-TZV	16.93	8.67	14.95	13.52
mPW3PBE/def2-TZVP	21.18	6.20	9.24	12.21
mPW3PBE/def2-TZVPD	28.55	10.11	8.48	15.71
ωB97XD/def2-TZV	34.59	9.39	15.44	19.81
ωB97XD/def2-TZVP	25.73	6.93	9.77	14.14
ωB97XD/def2-TZVPD	28.47	10.68	6.11	15.09

S4. Formation free energy values

Table S9. Enthalpy, entropy, and standard Gibbs free energy of formation calculated at the DLPNO-CCSD(T)/aug-cc-pVTZ//LC-*ω*HPBE/def2TZVP level.

	ΔH [kcal/mol]	ΔS [cal/(mol·K)]	ΔG(T=298.15 K) [kcal/mol]
1BuOH	0.000	0.000	0.000
2BuOH	-6.182	-35.388	4.537
3BuOH	-17.381	-78.151	6.290
4BuOH	-31.367	-120.152	5.027
5BuOH	-43.071	-163.062	6.320
6BuOH	-51.651	-203.558	10.006
7BuOH	-63.429	-246.272	11.166

Table S10. Enthalpy, entropy, and standard Gibbs free energy of formation calculated at the DLPNO-CCSD(T)/aug-cc-pVTZ//LC-ωHPBE/def2TZVP level.

	ΔH [kcal/mol]	ΔS [cal/(mol·K)]	ΔG(T=298.15 K) [kcal/mol]
1W	0.000	0.000	0.000
1BuOH	0.000	0.000	0.000
1Seed	0.000	0.000	0.000
1Seed1W	-15.236	-33.858	-4.981
1Seed2W	-28.029	-63.376	-8.833
1Seed3W	-46.078	-101.008	-15.483
1Seed4W	-63.989	-133.441	-23.570
1Seed5W	-79.125	-164.249	-29.374
1Seed1BuOH	-15.010	-43.518	-1.829
1Seed1BuOH1W	-32.738	-74.701	-10.111
1Seed1BuOH2W	-53.826	-113.055	-19.582
1Seed1BuOH3W	-67.890	-147.397	-23.243
1Seed1BuOH4W	-80.562	-174.789	-27.618
1Seed1BuOH5W	-101.432	-208.173	-38.376
1Seed2BuOH	-34.519	-86.420	-8.343
1Seed2BuOH1W	-54.876	-121.172	-18.174
1Seed2BuOH2W	-68.249	-157.647	-20.498
1Seed2BuOH3W	-84.625	-181.381	-29.685
1Seed2BuOH4W	-101.426	-226.328	-32.872
1Seed2BuOH5W	-116.165	-257.231	-38.250
1Seed3BuOH	-57.001	-132.353	-16.912
1Seed3BuOH1W	-72.276	-165.754	-22.071
1Seed3BuOH2W	-86.563	-199.694	-26.075
1Seed3BuOH3W	-100.940	-231.230	-30.901
1Seed3BuOH4W	-118.082	-270.082	-36.274
1Seed3BuOH5W	-129.592	-287.574	-42.485
1Seed4BuOH	-72.206	-174.431	-19.371
1Seed4BuOH1W	-88.882	-202.522	-27.538
1Seed4BuOH2W	-104.892	-242.513	-31.435
1Seed4BuOH3W	-116.783	-272.027	-34.386
1Seed4BuOH4W	-129.480	-295.701	-39.913
1Seed4BuOH5W	-149.975	-349.730	-44.043
1Seed5BuOH	-89.715	-218.145	-23.640
1Seed5BuOH1W	-102.033	-243.881	-28.162
1Seed5BuOH2W	-123.224	-296.014	-33.563
1Seed5BuOH3W	-135.302	-313.969	-40.201
1Seed5BuOH4W	-146.310	-351.437	-39.861
1Seed5BuOH5W	-162.553	-379.846	-47.498

Table S11. Enthalpy, entropy, and standard Gibbs free energy of formation calculated at the GFN2-*x*TB level (the low level of theory).

	$\Delta G(T=298.15\ K)$ [kcal/mol]		
	small seed	normal seed	big seed
1Seed	0.000	0.000	0.000
1Seed1BuOH	-9.626	-6.665	-11.634
1Seed2BuOH	-16.382	-12.959	-24.377
1Seed3BuOH	-23.006	-19.968	-31.528
1Seed4BuOH	-26.688	-23.749	-28.024
1Seed5BuOH	-32.000	-26.266	-34.953
	negative seed	neutral seed	positive seed
1Seed	0.000	0.000	0.000
1Seed1BuOH	-3.439	-6.665	-10.272
1Seed2BuOH	-6.247	-12.959	-21.748
1Seed3BuOH	-8.769	-19.968	-32.248
1Seed4BuOH	-10.965	-23.749	-39.852
1Seed5BuOH	-14.447	-26.266	-47.627

S5. Nucleation rate related tables

Table S12. Nucleation rates *J* and relative nucleation rates J/J_{ref} corresponding to Figure 7 in the main manuscript. Red color highlights the reference system.

T [K]	S [-]	J [$cm^{-3}s^{-1}$]	J/J_{ref}	S [-]	J [$cm^{-3}s^{-1}$]	J/J_{ref}
273.15	1	5.01E+10	0.21	5	6.18E+11	2.59
278.15	1	7.13E+10	0.30	5	9.13E+11	3.83
283.15	1	9.92E+10	0.42	5	1.32E+12	5.54
288.15	1	1.35E+11	0.57	5	1.87E+12	7.84
293.15	1	1.81E+11	0.76	5	2.59E+12	10.89
298.15	1	2.38E+11	**1.00**	5	3.53E+12	14.85
303.15	1	3.08E+11	1.29	5	4.73E+12	19.86

Table S13. Nucleation rates *J* and relative nucleation rates J/J_{ref} corresponding to Figure 8 in the main manuscript. Red color highlights the reference system.

T [K]	S [-]	J [$cm^{-3}s^{-1}$]	J/J_{ref}	S [-]	J [$cm^{-3}s^{-1}$]	J/J_{ref}
273.15	2.58	2.38E+11	1	14.65	2.38E+12	10
278.15	2.05	2.38E+11	1	10.40	2.38E+12	10
283.15	1.67	2.38E+11	1	7.70	2.38E+12	10

288.15	1.38	2.38E+11	1	5.94	2.38E+12	10
293.15	1.16	2.38E+11	1	4.72	2.38E+12	10
298.15	1.00	2.38E+11	**1**	3.85	2.38E+12	10
303.15	0.87	2.38E+11	1	3.23	2.38E+12	10

Table S14. Nucleation rates J and relative nucleation rates J/J_{ref} corresponding to Figure 11 in the main manuscript. Red color highlights the reference system.

RH [%]	S [-]	J [cm^{-3}s^{-1}]	J/J_{ref}	S [-]	J [cm^{-3}s^{-1}]	J/J_{ref}
0 ≈ 0.01	1	2.47E+11	**1.00**	5	3.63E+12	14.71
0.1	1	2.53E+11	1.03	5	3.64E+12	14.74
1	1	3.18E+11	1.29	5	3.71E+12	15.04
10	1	9.67E+11	3.92	5	4.43E+12	17.97
20	1	1.68E+12	6.82	5	5.22E+12	21.17
30	1	2.39E+12	9.69	5	6.00E+12	24.31
40	1	3.09E+12	12.54	5	6.77E+12	27.43
50	1	3.79E+12	15.36	5	7.53E+12	30.50
60	1	4.48E+12	18.17	5	8.28E+12	33.54
70	1	5.17E+12	20.95	5	9.02E+12	36.55
80	1	5.86E+12	23.73	5	9.76E+12	39.53
90	1	6.54E+12	26.48	5	1.05E+13	42.49
100	1	7.21E+12	29.23	5	1.12E+13	45.43

Table S15. Nucleation rates J and relative nucleation rates J/J_{ref} corresponding to Figure 13 (seed size part) in the main manuscript. Red color highlights the reference system.

$(NaCl)_y$	S [-]	J [cm^{-3}s^{-1}]	J/J_{ref}	S [-]	J [cm^{-3}s^{-1}]	J/J_{ref}
5	1	1.14E+12	0.81	5	5.72E+12	4.06
10	1	1.41E+12	**1.00**	5	7.06E+12	5.01
25	1	2.00E+12	1.42	5	1.00E+13	7.09

Table S16. Nucleation rates J and relative nucleation rates J/J_{ref} corresponding to Figure 13 (seed charge part) in the main manuscript. Red color highlights the reference system.

$(SEED)^q$	S [-]	J [cm^{-3}s^{-1}]	J/J_{ref}	S [-]	J [cm^{-3}s^{-1}]	J/J_{ref}
1-	1	7.97E+11	0.57	5	1.46E+13	10.34
0	1	1.41E+12	**1.00**	5	7.06E+12	5.01
1+	1	3.17E+12	2.25	5	1.59E+13	11.25

S6. Comparing low and high levels of theory

Throughout this discussion, we use the following shorthand for comparing two levels of theory:

Low level of theory (computationally affordable):

XTB = GFN2-*x*TB

High level of theory (computationally very expensive):

DLPNO = DLPNO-CCSD(T)/aug-cc-pVTZ//LC-ωHPBE/def2TZVP

Figure S5 shows the Gibbs formation free energies of $(NaCl)_{10}(BuOH)_{0\text{-}5}$ clusters calculated at the XTB and DLPNO levels of theory, computed using actual vapor concentrations. The low level of theory does not predict the existence of the modest energy barrier predicted by the higher-level methods, the energy difference between low and high level calculations is several kcal/mol. On the other hand, the trend is similar, and the energy differences become smaller when more butanol molecules are adsorbed onto the NaCl seed. Since the low level of theory calculations are significantly faster, we used this method for calculations of the butanol-seed system with various seed sizes and seed charges. We expect these calculations to provide qualitatively correct results.

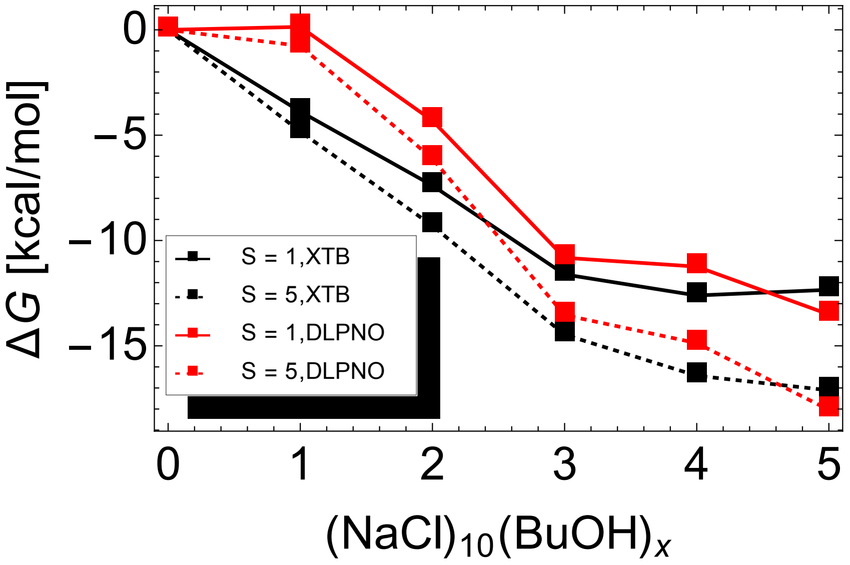

Figure S5. Gibbs free energies of formation computed using actual vapor concentrations. Red color represents calculations at the high level of theory (DLPNO), and black color represents calculations at the low level of theory (XTB). All calculations are performed at 25 °C and saturation ratios of 1 (solid lines) or 5 (dashed lines).

S7. Seed partial charges

To support the claim that small seeds have stronger Coulombic interactions due to a sharp corner in the seed geometry, we statistically analyzed the seed atomic partial charges. The analysis is performed just over Gibbs free energy global minimum structure obtained at the low level of theory (GFN2-*x*TB). The average value is taken over all seed chloride and sodium absolute partial charges, i.e. chloride partial charge is included without the minus sign. Figure S6 shows if only 0-2 butanol molecules are condensed on the seed, the absolute value of seed atomic partial charges of the small seed is greater than of the normal seed (or the large seed). The other effect lowering the cluster's Gibbs free energy is the interaction with butanol molecules. The overall behavior of partial charges of the normal and the small seed correlates with the Gibbs formation free energy difference shown in Figure S7.

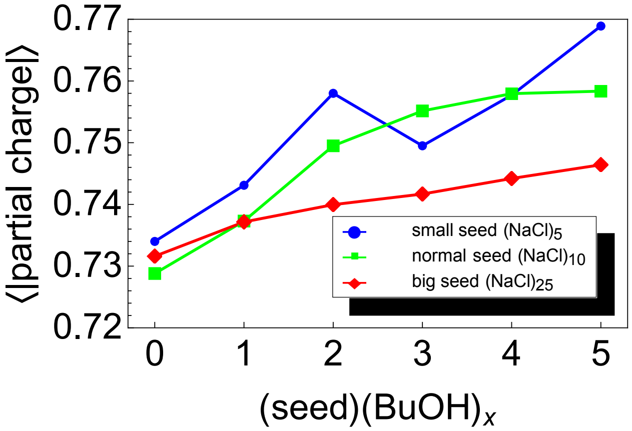

Figure S6. The seed average absolute atomic partial charge was calculated at the low level of theory (XTB). Various seed sizes are represented by a different color. Even though the partial charge of big seed is not important for this section, we added it only for illustration.

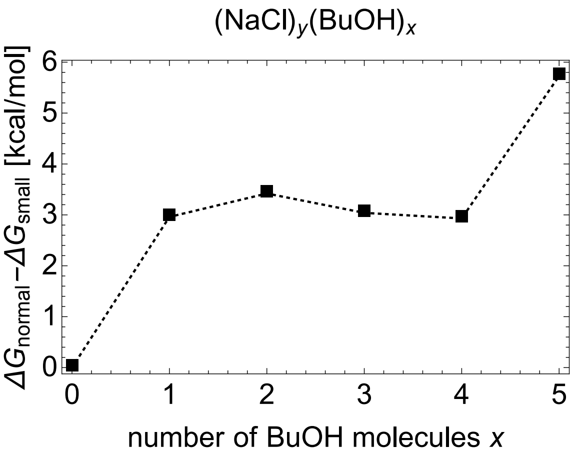

Figure S7. The difference between Gibbs formation free energies of normal and small seed shown in Figure 13a of the main text.

S8. Evolution of cluster concentrations

We examined all ACDC simulations for the time needed to reach a steady-state. Figure S8 shows the cluster concentration time evolution for the NaCl-BuOH-W system at 298.15 K, butanol saturation of 1, and humidity of 10 %. The nucleation rate at the steady-state for this condition is $\sim 10^{12}$ cm^{-3}s^{-1}. As mentioned in the main text, the only possible path for a cluster to grow into particles is via a 6th butanol molecule addition to the nucleating cluster. Figure S8 shows that the clusters containing five water molecules have a higher concentration in the steady-state as their growth via the addition of another water molecule is forbidden (i.e., the addition of a water molecule would lead to its immediate evaporation). The steady-state is due to this artifact reached after $\sim 10^{-6}$-10^{-5} s. Removing the barrier for outgrowing via water addition leads to the same nucleation rates, however, steady-state is achieved in shorter time $<10^{-7}$ s. The reason why we forbid outgrowing via water is explained in the main text. Additionally, the steady-state is achieved faster when both monomer concentrations are increased, or temperature is lowered. In all cases, the steady-state is reached several orders of magnitude faster than typical times between seed injection to the condensation chamber and its detection ($\sim$100 ms-1 s).

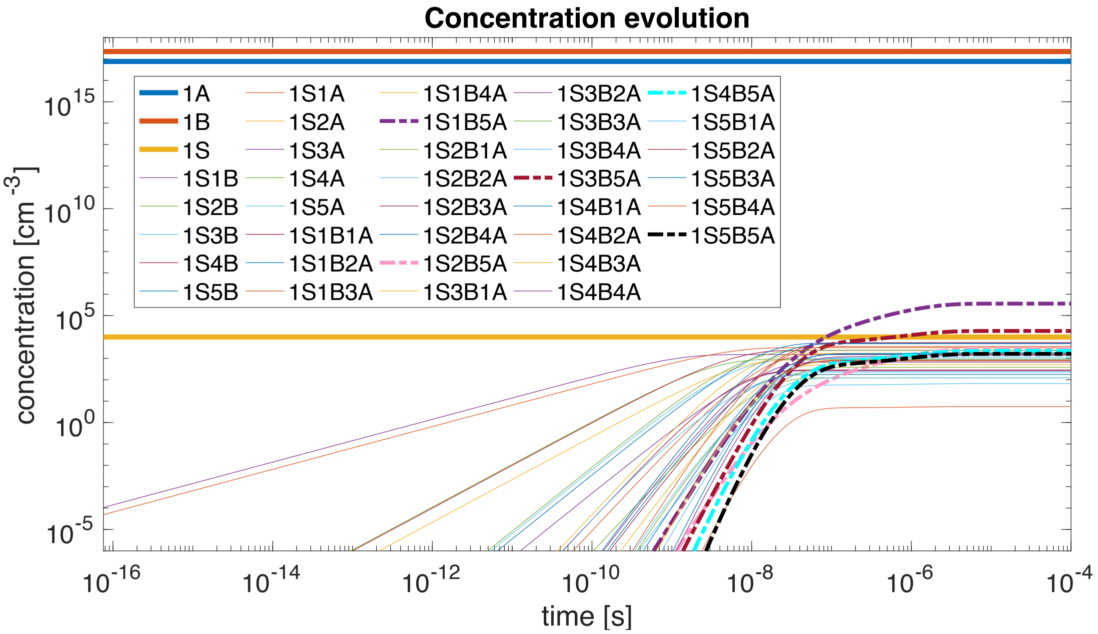

Figure S8. Monomer and cluster concentration evolution in time until the steady-state. This figure has been plotted for simulation of $(NaCl)_{10}$-BuOH-W system at 298.15 K with the constant concentration: C(1S) = 10^4 cm^{-3}, C(1W) = $3.1\cdot10^{16}$ cm^{-3} (10 % humidity), and C(1B) = $2.4\cdot10^{17}$ cm^{-3} (S = 1), where S = NaCl seed, W = water molecule, and B = butanol molecule.

References

[1] Moc, J.; Simmie, J. M.; Curran, H. J. The elimination of water from a conformationally complex alcohol: A computational study of the gas phase dehydration of n-butanol. *J. Mol. Struc.* **2009**, 928, 149–157.

[2] Bannwarth, C.; Ehlert, S.; Grimme, S. GFN2-*x*TB—An Accurate and Broadly Parametrized Self-Consistent Tight-Binding Quantum Chemical Method with Multipole Electrostatics and Density-Dependent Dispersion Contributions. *J. Chem. Theory Comput.* **2019**, 15, 1652–1671.

[3] Grimme, S.; Bannwarth, C.; Shuskov, P. A robust and accurate tight-binding quantum chemical method for structures, vibrational frequencies, and noncovalent interactions of large molecular systems parametrized for all spd-block elements (Z = 1-86). *J. Chem. Theory Comput.* **2017**, 13, 1989–2009.

[4] Kubečka, J.; Besel, V.; Kurtén; Myllys, N.; Vehkamäki, H. Configurational Sampling of 4oncovalent (Atmospheric) Molecular Clusters: Sulfuric Acid and Guanidine. *J. Phys. Chem. A* **2019**, 28, 6022–6033.

[5] Frisch, M. J.; Trucks, G. W.; Schlegel, H. B.; Scuseria, G. E.; Robb, M. A.; Cheeseman, J. R.; Scalmani, G.; Barone, V.; Petersson, G. A.; Nakatsuji, H. et al. Gaussian 16 Revision A.03. **2016**; Gaussian Inc. Wallingford CT.

[6] Funes-Ardois, R. P. GoodVibes 1.0.1.

[7] Neese, F. The ORCA program system. Wiley Interdiscip. Rev.: *Comput. Mol. Sci.* **2012**, 2, 73–78.

[8] Haynes, W. M.; Lide, D. R.; Bruno, T. J. CRC handbook of chemistry and physics: aready-reference book of chemical and physical data (97th Edition); Boca Raton, Florida; CRC Press, **2016**.
